# Multibeam Reflectarrays in Ka-Band for Efficient Antenna Farms Onboard Broadband Communication Satellites †

**DOI:** 10.3390/s21010207

**Published:** 2020-12-31

**Authors:** Daniel Martinez-de-Rioja, Eduardo Martinez-de-Rioja, Yolanda Rodriguez-Vaqueiro, Jose A. Encinar, Antonio Pino

**Affiliations:** 1 Information Processing and Telecommunications Center, Universidad Politécnica de Madrid, 28040 Madrid, Spain; jose.encinar@upm.es; 2 Area of Signal Theory and Communications, Universidad Rey Juan Carlos, 28942 Madrid, Spain; eduardo.martinez@urjc.es; 3 AtlanTTic Research Center, Universidade de Vigo, 36310 Vigo, Spain; yrvaqueiro@com.uvigo.es (Y.R.-V.); agpino@com.uvigo.es (A.P.)

**Keywords:** reflectarray antennas, multibeam antennas, dual band reflectarrays, communication satellites, Ka-band

## Abstract

Broadband communication satellites in Ka-band commonly use four reflector antennas to generate a multispot coverage. In this paper, four different multibeam antenna farms are proposed to generate the complete multispot coverage using only two multibeam reflectarrays, making it possible to halve the number of required antennas onboard the satellite. The proposed solutions include flat and curved reflectarrays with single or dual band operation, the operating principles of which have been experimentally validated. The designed multibeam reflectarrays for each antenna farm have been analyzed to evaluate their agreement with the antenna requirements for real satellite scenarios in Ka-band. The results show that the proposed configurations have the potential to reduce the number of antennas and feed-chains onboard the satellite, from four reflectors to two reflectarrays, enabling a significant reduction in cost, mass, and volume of the payload, which provides a considerable benefit for satellite operators.

## 1. Introduction

**Figure 1:**
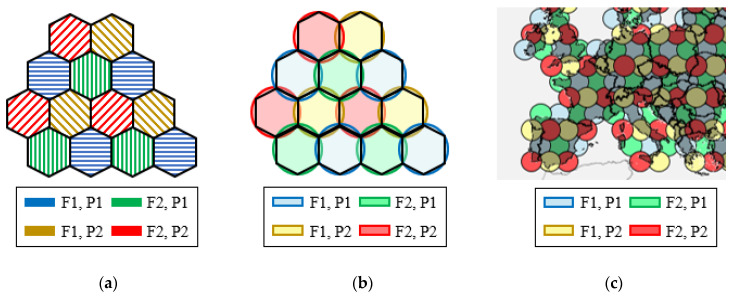
Example of a four-color multispot coverage. (**a**) Hexagonal service cells working in four different colors. (**b**) Hexagonal cells covered by circular beams generated in two orthogonal polarizations (P1, P2) and two frequencies (F1, F2). (**c**) Example of a European four-color coverage that would be generated by a geostationary satellite.

In the past years, the continental contoured beams traditionally used for broadcast satellite applications in Ku-band are being replaced by cellular coverages to provide broadband services typically in Ka-band [1]. The cellular coverages are formed by between 50 and 100 slightly overlapping spot beams, generated with a frequency and polarization reuse scheme of four colors, where each color is associated to a unique combination of frequency and polarization [2]. Thus, the four-color scheme requires splitting the available user spectrum into two different frequency sub-bands and two orthogonal circular polarizations (CP) [2,3]. The generation of four-color coverages makes it possible to spatially isolate the spots generated with same frequency and polarization, so the interference between spots is reduced and the throughput of the users can be increased without modifying the operational bandwidth of the system. The high-gain beams generated by the satellite antennas produce circular spots on the Earth’s surface. The directions of the beams are adjusted to produce a triangular lattice of circular spots. Therefore, the service area can be split into hexagonal cells, each of which is covered by a circular beam [2]. Figure 1 shows an example of a four-color coverage based on hexagonal cells. The diameter of the spots is defined depending on the capacity need of the system. Typically, the spots cover a circular area of around 250–300 km in diameter, which corresponds to a beamwidth of about 0.5–0.65° for the beams produced by the satellite antennas [3].

The generation of the multispot coverage from the satellite is typically accomplished by using four multi-fed single offset reflectors operating by a single feed per beam (SFPB) architecture [3,4]. Reflectors offer a reliable and simple operation; however, they cannot generate the beams with an angular separation between adjacent beams as small as required for this application, since overlapping feeds would be required. Thus, four multi-fed reflectors are usually employed onboard the satellite, where each reflector produces all the beams in a specific frequency and polarization, which are spatially isolated from each other. In this way, each reflector generates the beams associated to one color in a lattice of non-contiguous spots (the spots are separated by gaps that will be covered by the beams generated by the other reflectors). Therefore, the interlaced beams produced by the four reflectors form the final four-color cellular coverage of contiguous spots.

Due to the severe constraints in weight and volume of satellites, the use of four reflectors can be seen as a suboptimal antenna farm. Different multibeam antenna solutions have been proposed lately to reduce the number of antennas onboard the satellite [5]. The use of lenses [6] or array antennas [7] reduces the radiation efficiency and increases the complexity of the antenna architecture, while the highly oversized reflector proposed in [8] comprises a prohibitive stowage volume. In this paper, four different multibeam antenna solutions are proposed based on reflectarray antennas [9], following the study introduced in [10]. Each multibeam reflectarray is intended to generate half the required multispot coverage, making it possible to reduce the number of antennas onboard the geostationary satellite from four reflectors to two reflectarrays. The antenna specifications commonly required in real scenarios (described in Section 2) will be used to analyze the performance of the proposed reflectarrays. In Section 3, a 1.8 m flat reflectarray and a 1.8 m parabolic reflectarray will be proposed to generate a four-color coverage only for the transmission link with a single antenna aperture. Then, a dual-reflectarray system and a 1.8 m parabolic reflectarray will be shown in Section 4 and Section 5, respectively, to generate half the required spots (two colors) simultaneously in transmission (Tx) and reception (Rx). The main characteristics of the four reflectarrays will be compared in Section 6, proving the great potential of reflectarrays for multibeam satellite applications and their capability to halve the number of antennas and feed-chains required onboard the satellite to generate a four-color coverage.

## 2. Mission Scenario and Requirements of the Antenna System

**Figure 2:**
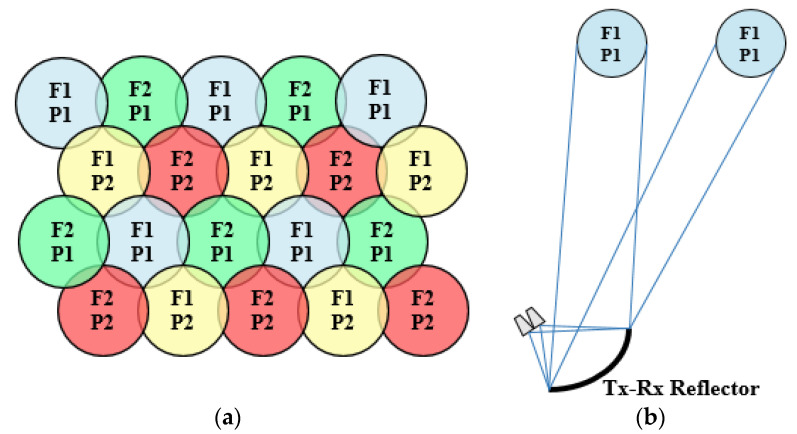
(**a**) Four-color multispot coverage required simultaneously in Tx and Rx. (**b**) Operating principle of current reflectors used onboard the satellite, operating simultaneously in Tx and Rx.

The main specifications of the cellular coverage, shown in Table 1, have been established from those of current multibeam satellites in Ka-band [2,3,11,12] and the Rec. ITU-R S.672-4 [13]. The coverage must be formed by around 100 spots in a triangular lattice, generated with a four-color reuse scheme based on two different frequencies and two orthogonal CP. The four-color coverage must be simultaneously generated at Tx and Rx user frequencies in Ka-band, which comprise from 19.2 to 20.2 GHz for Tx and from 29.0 to 30.0 GHz for Rx. Thus, both Tx and Rx frequency bands must be divided into two sub-bands to provide the four-color coverage with two orthogonal CP simultaneously in Tx and Rx. Figure 2 shows a schematic representation of a multispot coverage with a four-color reuse scheme of two frequencies (F1, F2) and two polarizations (P1, P2) together with the operating scheme of the current multi-fed reflectors used onboard the satellite, where each reflector generates a lattice of non-contiguous spots in a single color, simultaneously in Tx and Rx (the interlaced beams produced by the four reflectors form the final four-color coverage of contiguous spots). The diameter of the spots is set to 0.65° to cover a circular area of around 300 km on the Earth. The angular separation between adjacent spots from center to center to form the appropriate triangular lattice of spots must be 0.56° (computed as sin(60°)·0.65°). The minimum end of coverage (EOC) gain of the beams is set to 45 dBi, the minimum single-entry carrier over interference ratio (C/I) at 20 dB, and the minimum co-polar over cross-polar discrimination (XPD) is also fixed to 20 dB.

**Figure 3:**
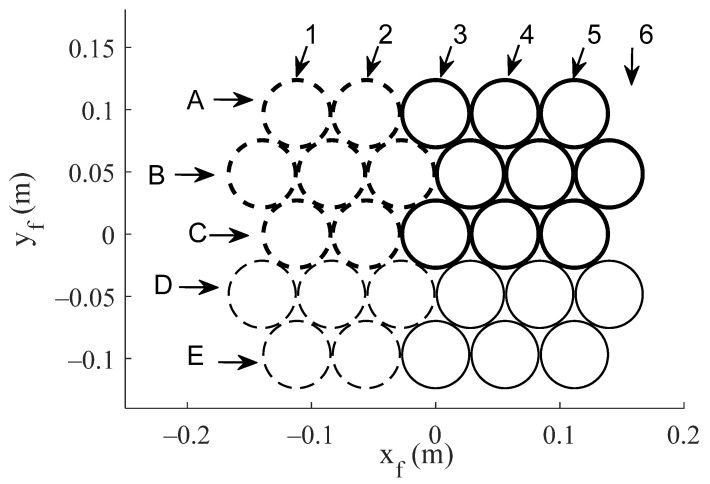
Cluster of 27 feeds defined to illuminate the different antenna farms.

The proposed antenna configurations operate by an improved SFPB configuration, taking advantage of the ability of reflectarrays to generate independent beams at different frequencies [14] or polarizations [15] with a single feed. The proposed reflectarrays will be illuminated by a cluster of 27 feeds defined with a triangular lattice, in order to provide the required multibeam coverage with a triangular grid of spots. The feeds have been modelled considering the 54 mm Ka-band feed-chain reported in [4], thus the separation between adjacent feeds has been set to 55 mm. The radiation pattern of the feeds has been modelled in the simulations by an ideal cos^q^(*θ*) distribution. Figure 3 shows the proposed cluster of 27 feeds using the local coordinate system (x_f_, y_f_), the origin of which matches the center of the aperture of the central feed (C3 in Figure 3). The symmetry of each antenna configuration will be used to reduce the number of feeds considered in simulations (the lines of feeds A, B, C, plotted with thicker lines, or the array of 3x5 feeds plotted with solid lines in Figure 3).

## 3. Antenna Farm Based on Two Single-Band Reflectarrays

**Figure 4:**
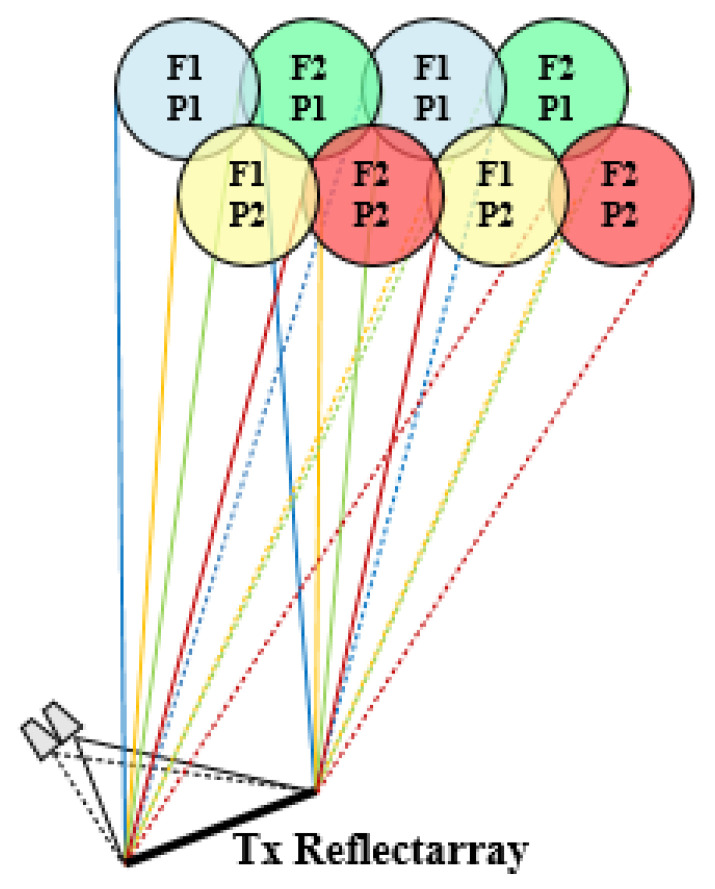
Operating principle of the proposed single-band reflectarray.

The first strategy introduced in [10] to generate a complete four-color multispot coverage simultaneously in Tx and Rx is based on the design of a reflectarray antenna to generate four spaced beams per feed in two different operating frequencies and two orthogonal polarizations (i.e., four spaced beams in four different colors). In this way, the limitation of conventional reflectors to provide such closely spaced beams is overcome by the generation of four adjacent beams per feed. Thus, a reflectarray illuminated by the proposed cluster of 27 feeds would generate a complete four-color coverage of 108 spots only for a single band (Tx or Rx). To provide multispot coverage both in Tx and Rx, two single-band reflectarrays designed with the same technique to produce four adjacent beams per feed would be required onboard the satellite (one for Tx and the other for Rx). The operating scheme of the proposed solution is shown in Figure 4 for a flat reflectarray to operate in the Tx band in Ka-band.

**Figure 5:**
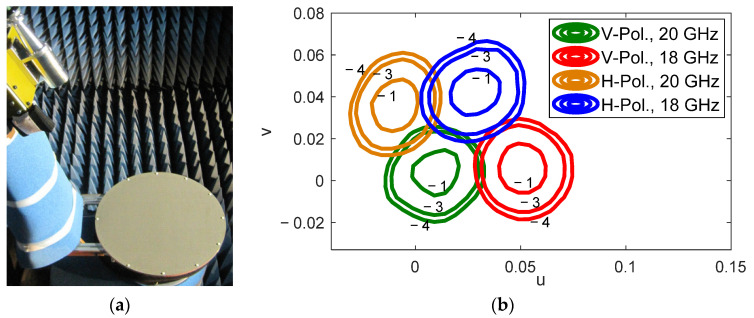
Reflectarray prototype to generate four spaced beams per feed [17]. (**a**) Picture of the prototype, (**b**) measured pattern contours at −1, −3, and −4 dB of the four beams generated by the same feed.

This solution requires a reflectarray with independent operation at close frequencies (within the same band for Tx or Rx in Ka-band) and also independent operation in orthogonal polarizations, which increases the complexity of the reflectarray cells and the design technique. The controlled application of the beam squint effect with frequency has been used to reduce the design complexity of the reflectarray antenna, as shown in [16]. The method to generate four spaced beams in four different colors per feed has been experimentally validated in [17] for a 43 cm reflectarray antenna. The prototype operates in linear polarization (LP), but the same design technique can be applied to produce the beams in CP by an appropriate selection of the reflectarray cells. Figure 5 shows the 43 cm prototype in the anechoic chamber and the measured radiation patterns of the four spaced beams in four different colors, according to the normalized angular coordinates u = sin*θ*·cos*φ*, v = sin*θ*·sin*φ*.

Two different approaches have been evaluated to produce a multispot coverage following the design technique developed in [17]: first, using a flat reflectarray as proposed in [10], and second, using a reflectarray with a parabolic surface. The two reflectarrays are expected to generate a four-color coverage for Tx in Ka-band using the feed cluster shown in Figure 3. Due to the symmetry of the antenna system, the simulations have considered the lines of feeds A, B, and C in Figure 3 (plotted with thicker lines in Figure 3), since there will be minimal differences between the beams generated by the lines of feeds B, C and D, E. The two reflectarrays have a diameter of 1.8 m and operate at 19.45 and 19.95 GHz in orthogonal polarizations. The simulations have considered ideal reflectarray elements to provide the required phase-shift in each color without phase errors or ohmic losses. The preliminary results will be used to evaluate the strengths and weaknesses of the design method. The conclusions reached in this study can also be applied to the associated reflectarrays used for Rx, since the design method would be identical.

### 3.1. Flat Transmit Reflectarray to Generate Four Spaced Beams per Feed in Ka-Band

**Figure 6:**
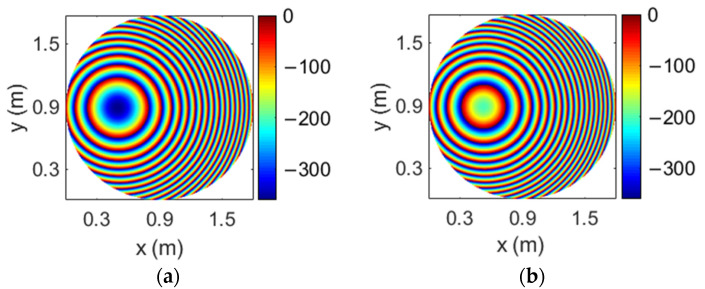
Required phase distributions of the 1.8 m flat reflectarray at the (**a**) lower and (**b**) upper operating frequencies for the same CP.

A flat reflectarray has been proposed to generate four spaced beams per feed according to the design technique shown in [17]. The reflectarray has a diameter of 1.8 m and it is formed by 44125 reflectarray cells arranged in a 239 × 235 lattice, the period of which has been set to 7.5 mm to avoid grating lobes. As a result of the design method, four different phase distributions are implemented in the reflectarray surface (one for each combination of frequency and polarization). Figure 6 shows the required phase distributions at 19.45 and 19.95 GHz for one CP (there are no appreciable differences with the phase distributions for the orthogonal CP). The large size of the reflectarray and its flat configuration are the reason why the phase distributions depicted in Figure 6 show a large number of 360° cycles. The abrupt variations in the phase distributions limit the antenna performance and reduce the operational bandwidth.

**Figure 7:**
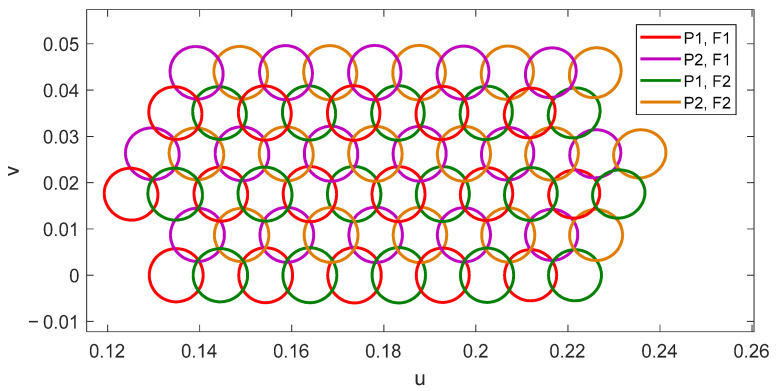
Simulated 46 dBi pattern contours of the 64 beams generated by 16 feeds in four different colors with a 1.8 m flat reflectarray.

The results of the conducted simulations when the 1.8 m reflectarray is illuminated by the 16 feeds placed in the lines A, B, and C in Figure 3 are shown in Figure 7, which presents the pattern contours of the beams for a 46 dBi gain. The simulated pattern contours show a coverage of 64 beams generated in a four-color reuse scheme. The distribution of beams meets the required triangular lattice of spots, as well as the separation and diameter of the circular spots (0.56° and 0.65°, respectively).

**Figure 8:**
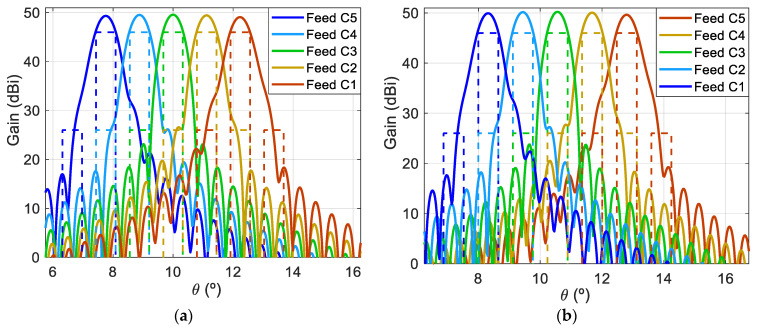
Cut of the simulated radiation patterns in v = 0 for the beams generated at the (**a**) lower and (**b**) upper frequency in the same circular polarization (CP).

The main cuts of the radiation patterns have been evaluated to estimate the interferences between beams generated in the same color. Figure 8 shows the horizontal cut of the radiation patterns in the plane v = 0 for the beams generated at both frequencies. Due to the monofocal antenna design, the extreme beams of the coverage show some aberrations that increase the C/I. Since the side-lobe levels of the beams can be reduced by slightly increasing the antenna aperture and reducing the edge illumination, this drawback can be easily overcome (space reflectors in Ka-band usually have a diameter of 2.3 m). Thus, the diameter of 1.8 m has been maintained for the rest of the reflectarray antennas proposed in this paper, expecting similar C/I levels. The use of ideal reflectarray cells prevents an accurate characterization of the cross-polar radiation, which is mainly produced by the reflectarray elements and the offset antenna configuration. Previous reflectarray prototypes with offset configurations have provided XPD above 25 dB [17,18].

The main constraint of this strategy is the operational bandwidth of the reflectarray, limited by the abrupt phase variations shown in Figure 6 and the independent operation at two close frequencies within the same link (Tx or Rx) in Ka-band.

### 3.2. Parabolic Transmit Reflectarray to Generate Four Spaced Beams per Feed in Ka-Band

The design of large and flat reflectarrays results in inappropriate phase distributions (with fast phase variations) that limit the antenna performance. The previous design of a multibeam reflectarray to generate four spaced beams per feed has been improved to fit a 1.8 m parabolic reflectarray. In this configuration, the parabolic surface shapes the beams as a conventional reflector, while the reflectarray elements only introduce a small phase correction to slightly deviate the beams produced in orthogonal polarizations and/or at different operating frequencies.

**Figure 9:**
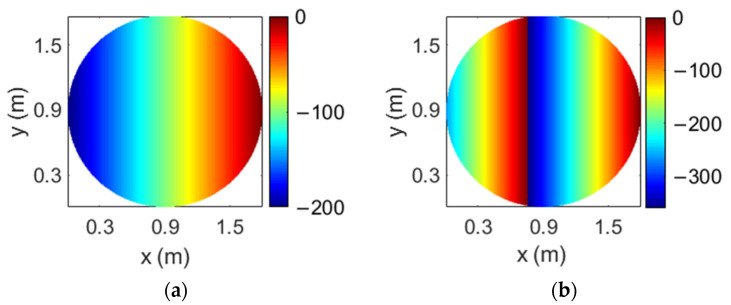
Required phase distributions of the 1.8 m parabolic reflectarray at the (**a**) lower and (**b**) upper operating frequencies for the same CP.

The four required phase distributions that must be implemented in the parabolic reflectarray have been computed and Figure 9 shows the required phase distributions at 19.45 and 19.95 GHz for one CP (the phase distributions for the orthogonal CP have a similar behavior). The phase distributions in Figure 9 show a great improvement with respect to the equivalent distributions for a flat reflectarray (see Figure 6), presenting smooth phase variations and a reduced number of 360° cycles. The 1.8 m parabolic reflectarray antenna has been simulated considering the same cluster of feeds as in the previous design, producing a four-color coverage of 64 beams similar to that shown in Figure 7. There are no appreciable differences between the simulated radiation patterns of both flat and parabolic reflectarrays (a comparison between the antenna performances of flat and parabolic reflectarrays to produce four beams per feed can be seen in detail in [19]).

The results obtained for this (flat or parabolic) antenna farm show that a single reflectarray with 27 feeds would produce a four-color coverage of 108 beams only in the Tx or Rx link of the Ka-band, so two reflectarrays would be needed to generate a complete multispot coverage both in Tx and Rx. As a result, the number of antennas would be halved with respect to the conventional four-reflector configuration, and the same would happen with the number of feed-chains (since each feed produces four beams in Tx or Rx).

Moreover, this solution makes it possible to reduce the complexity of the feed-chains, since each reflectarray would be illuminated by single-band feeds, instead of the current dual-band feed-chains [4]. The use of independent reflectarrays to operate in Tx and Rx can be exploited to reduce the size of the Rx antenna, due to its higher operating frequency. However, the main limitation is related to the independent operation at two close frequencies. Although the parabolic surface has overcome the drawback associated with the required phase distributions, further research is needed on the reflectarray cell that should be used in a real scenario to achieve dual-band operation at near frequencies. The cell proposed in [20] could be scaled to operate at two close frequencies within the Tx band in Ka-band in dual CP. Once the reflectarray cell is defined, intense optimizations should be applied to provide an independent operation in both frequencies with a stable performance in band.

## 4. Antenna Farm Based on Two Dual-Band Dual Reflectarray Configurations

The constraints arising from the design of reflectarrays with independent operation at two close frequencies (within the same band for Tx or Rx) can be overcome by implementing the dual-frequency operation in two separate frequency bands. Then, the reflectarray must generate two spaced beams per feed in orthogonal CP simultaneously at Tx and Rx frequencies in Ka-band. In this way, the reflectarray is expected to generate all the beams in two different colors simultaneously in Tx and Rx. A second dual-band reflectarray with slightly different operating frequencies would generate the second half of the coverage, and the interlaced coverages of the two reflectarrays would form the complete four-color coverage in Tx and Rx. However, the design of a reflectarray with independent operation in dual CP simultaneously at two frequency bands is a complex task.

**Figure 10:**
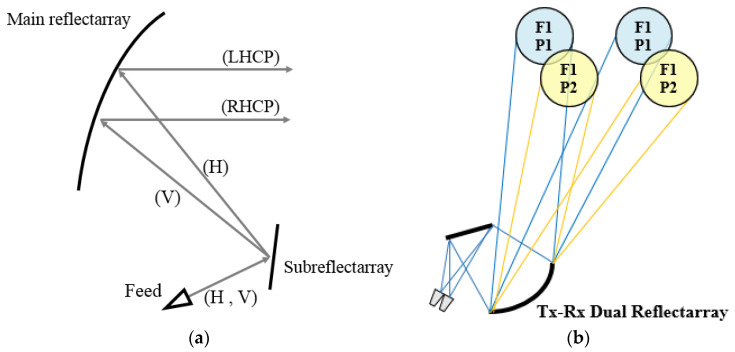
Dual reflectarray system proposed to operate simultaneously in Tx and Rx. (**a**) Operating principle of the dual-configuration, (**b**) functional scheme of the proposed antenna on the satellite.

In this strategy, the reflectarray has been defined with a dual-antenna configuration based on a flat subreflector reflectarray and a main parabolic reflectarray. The use of a dual configuration makes it possible to simplify the implementation of the dual-band operation in dual CP thanks to the larger number of degrees of freedom provided by the use of two reflectarrays. In this way, the feeds will operate in dual-LP, which implies a lesser complexity of the feed-chain than in the case of dual-CP operation (there is no need for a polarizer in the feed-chain). Then, the subreflectarray will be designed to deviate (without focusing) the dual-LP beams radiated by the feeds by ±0.28° (in opposite directions for each LP) simultaneously in Tx and Rx. The main reflectarray will be designed to convert the incident dual-LP field into dual-CP, at the same time as its parabolic surface focuses high-gain beams. Figure 10a shows the operating principle of the dual reflectarray antenna to generate two spaced beams in left-handed and right-handed CP (LHCP and RHCP, respectively) from a single feed operating in horizontal and vertical LP (H and V, respectively). Figure 10b shows the operating scheme of the proposed antenna to generate half the required multispot coverage (two colors) simultaneously in Tx and Rx.

The operating principles behind this solution have been experimentally validated separately. The design of a dual-band reflectarray with independent operation in dual LP at two separated frequencies can be reached in a direct way, as proposed in [21], by means of reflectarray cells based on orthogonal sets of parallel dipoles for each band. A polarizing reflectarray to convert dual LP into dual CP with broadband operation from 20 to 30 GHz (covering both Tx and Rx bands) has been demonstrated in [22].

**Figure 11:**
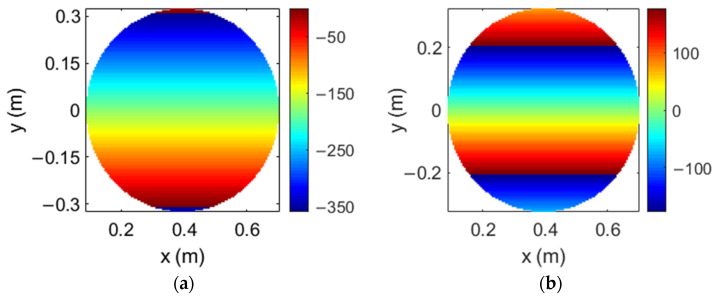
Required phase distributions of the 0.65 m flat subreflectarray at the (**a**) lower and (**b**) upper operating frequencies for the same linear polarization (LP).

The dual reflectarray designed to produce multiple spot beams in Ka-band has been defined on the basis of a Cassegrain system. The flat subreflectarray has a diameter of 0.65 m and it is formed by 11,497 cells disposed in a 117 × 125 grid, considering the same type of reflectarray cells used in [23] (based on two orthogonal set of parallel dipoles) with a period of 5.3 mm. The main parabolic reflectarray has a diameter of 1.8 m, formed by 62,654 cells disposed in a 286 × 279 grid. It has been designed using the polarizing cells shown in [22] (based on three coplanar parallel dipoles) with a period of 5 mm. In contrast to the complex phase distributions computed for the previous flat 1.8 m reflectarray (see Figure 6), the flat subreflectarray only deviates the orthogonal LP beams radiated by the feeds in opposite directions, so the required phase distributions present a smooth phase variation at both frequencies. Figure 11 shows the phase distributions implemented in the subreflectarray at 19.7 GHz and 29.5 GHz in one LP; note that the phase distributions in the orthogonal LP show the opposite phase variation at each frequency to deviate the orthogonal LP beam in the opposite direction. Then, the main parabolic reflectarray only converts the dual-LP incident field into dual-CP, so their cells have been designed to introduce a phase difference of 90° between the two orthogonal components of each incident LP [22].

**Figure 12:**
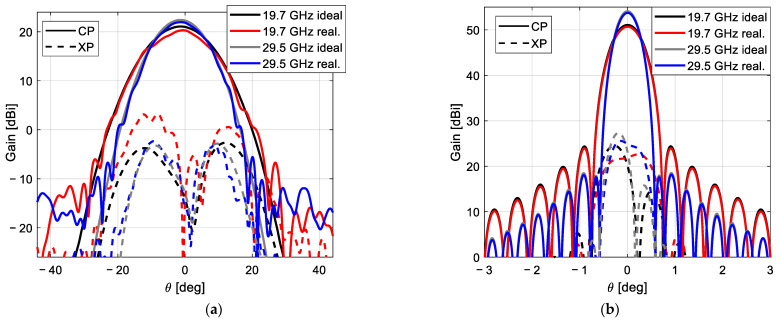
Simulated radiation patterns at 19.7 and 29.5 GHz in the azimuth plane of the ideal and realized antenna components. (**a**) Subreflectarray for H polarization, (**b**) main reflectarray for right-handed circular polarizations (RHCP).

The design of the subreflectarray and the main parabolic reflectarray has been carried out separately. The radiation patterns of the designed subreflectarray (without the main reflectarray) have been simulated and the results have been compared with those obtained considering ideal reflectarray cells (providing the required phase distributions without any phase errors and zero dielectric losses). As an example, Figure 12a shows the comparison between the ideal and realized patterns in the azimuth plane (φ = 90°) for the subreflectarray in H polarization at 19.7 and 29.5 GHz. Note that the patterns in Figure 12a show a wide main lobe since the subreflectarray does not focus the beam radiated by the feed (the parabolic surface of the main reflectarray will focus the beam). In the same way, the simulated radiation patterns of the designed polarizing main reflectarray (without the subreflectarray, so that the main reflectarray is illuminated from the virtual focus of the dual system) have been computed and compared with the simulations performed with ideal cells. Figure 12b shows the comparison between the ideal and realized patterns in the azimuth plane at 19.7 and 29.5 GHz for the polarizing main reflectarray in RHCP (obtained from the H polarization radiated by the feed).

The patterns in Figure 12 show a satisfactory agreement between the designed and ideal performances of both reflectarrays. The dual system configuration results in relatively large incidence angles from the feed on the subreflectarray, which complicates the design of the elements of the subreflectarray. As a result, the simulated patterns of the designed subreflectarray show a slightly lower gain and higher cross-polar levels than in the ideal case (see Figure 12a). On the other hand, the simulated results of the designed main reflectarray show an excellent performance, which is practically identical to the ideal behavior (see Figure 12b).

**Figure 13:**
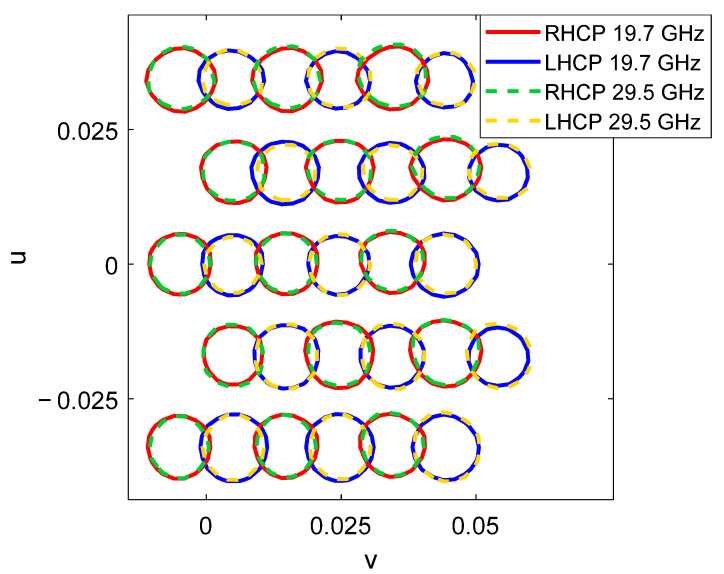
Simulated pattern contours for the 30 beams generated by 15 feeds illuminating the proposed dual reflectarray at 19.7 GHz and 29.5 GHz.

Finally, the dual antenna configuration is illuminated by the cluster of 27 feeds shown in Figure 3 rotated 90° in the x_f_y_f_-plane. The simulations of the complete dual reflectarray have considered 15 of the 27 feeds (an array of 3 × 5 feeds, plotted with solid lines in Figure 3), since the beams produced by the remaining feeds are expected to have similar behavior. The simulated pattern contours of the 30 beams generated by the designed dual reflectarray simultaneously at 19.7 GHz and 29.5 GHz are depicted in Figure 13 at the EOC levels of the beams (between 45 and 46 dBi). The simulated beams match with the required spot distribution, where there is room for the beams that would be generated by a second dual reflectarray operating at slightly different frequencies to form the complete four-color multispot coverage in Tx and Rx.

**Figure 14:**
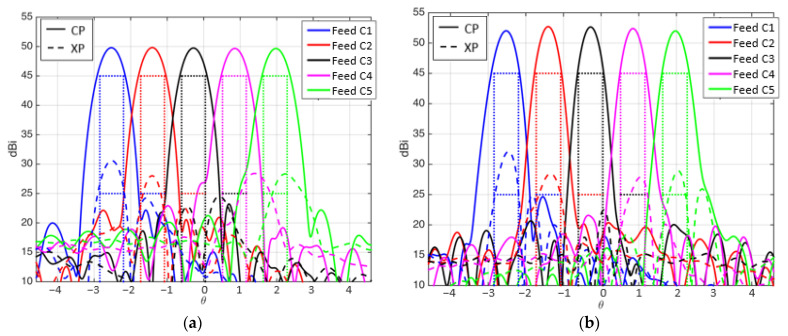
Cut of the simulated radiation patterns in u = 0 for the beams generated at (**a**) 19.7 GHz and (**b**) 29.5 GHz in same CP.

The main cuts of the radiation patterns have revealed similar C/I levels as those of the previous 1.8 m reflectarray. Figure 14 shows the cut of the radiation patterns in the plane u = 0 for the beams produced by the central row of feeds at both frequencies. The simulated cross-polar radiation provides an XPD lower than 20 dB for some beams. The analysis of the results has shown that the increase of the cross-polar component is mainly produced in the subreflectarray, so an optimization procedure should be applied to the dual-band dual-LP cells to reduce the cross-polarization. Moreover, the use of a dual configuration could be used to implement a bifocal technique to improve the shaping of the beams generated in the edge of the coverage [24].

In conclusion, the simulated results of the dual reflectarray configuration prove the capability of the proposed multibeam antenna to generate half the required four-color coverage simultaneously in Tx and Rx. The dual-band operation involving separate frequencies simplifies the design of the reflectarray elements, while the dual configuration simplifies the process of generating two spaced beams in orthogonal CP per feed, splitting it into two straightforward stages (deviation of the LP beams in opposite directions and dual-LP to dual-CP conversion), the principles of which have been experimentally validated previously. The simulated results show that a further optimization is required for the subreflectarray to improve the XPD, although the flexibility provided by the dual configuration could be also used to improve the overall performance of the antenna.

## 5. Antenna Farm Based on Two Dual-Band Offset Parabolic Reflectarrays

Bearing in mind the better performance of the previous dual-band dual reflectarray with respect to the single-band reflectarray, the final proposed antenna farm is also based on the use of a dual-band reflectarray to generate all the beams associated to two colors simultaneously in Tx and Rx. In this case, a single offset parabolic reflectarray is proposed instead of the previous dual configuration, reducing the number of components in the antenna configuration.

**Figure 15:**
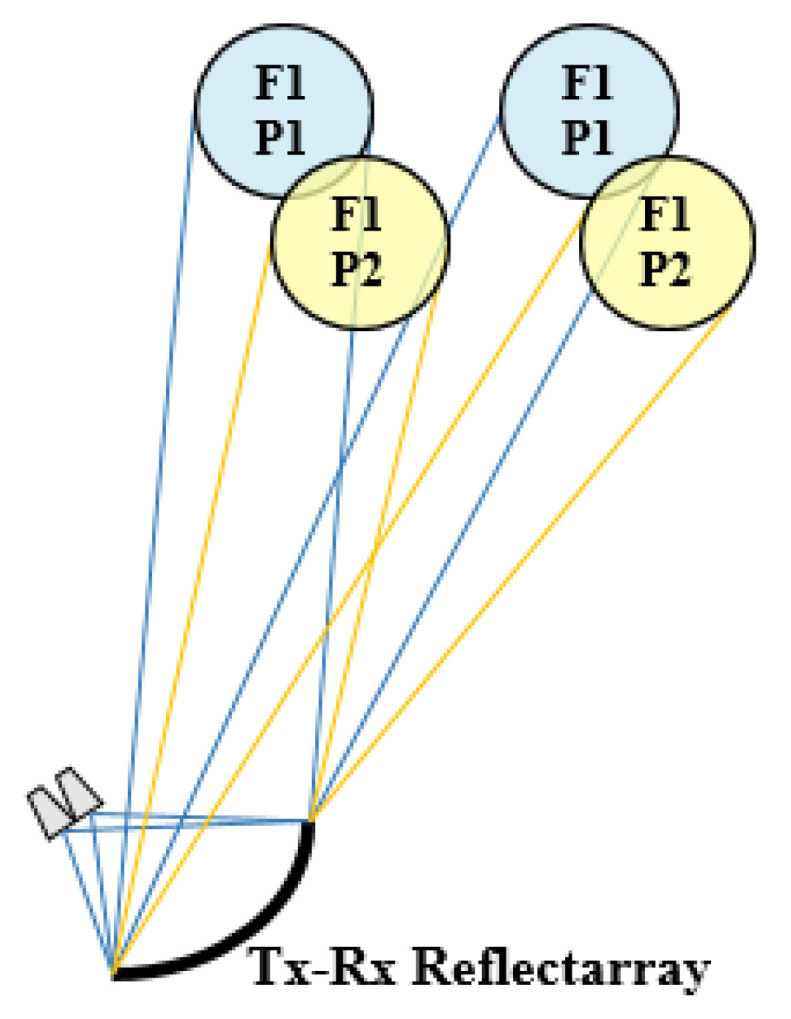
Operating principle of the proposed single-band parabolic reflectarray.

The generation of two spaced beams in orthogonal CP per feed is now completely managed by the reflectarray elements placed on the parabolic reflectarray surface. The operating principle of the antenna is based on the concept proposed in [25], where a single-band parabolic reflectarray deviates the orthogonal CP beams focused by the parabolic surface in opposite directions by means of the variable rotation technique (VRT) applied to the reflectarray elements. In our solution, a dual-band offset parabolic reflectarray is proposed to focus the beams by its parabolic surface and deviate the orthogonal CP by the application of VRT simultaneously at Tx and Rx frequencies. The dual-band operation by VRT is achieved using the reflectarray cells proposed in [20]. As a result, a single offset parabolic reflectarray illuminated by dual-CP feeds generates two spaced beams in orthogonal CP per feed at Tx and Rx frequencies in Ka-band. The operating scheme of the proposed reflectarray to generate half the required four-color coverage is shown in Figure 15.

**Figure 16:**
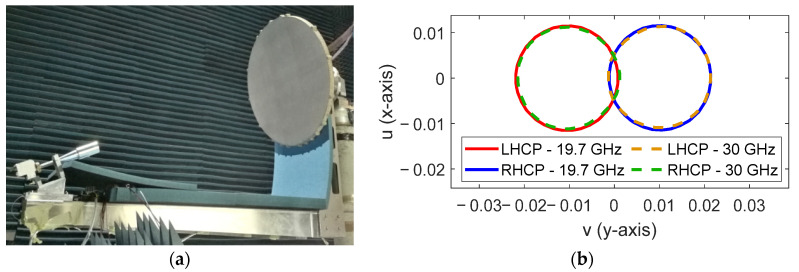
Dual band parabolic reflectarray prototype to generate two spaced beams per feed [26]. (**a**) Picture of the prototype, (**b**) measured 40.6 dBi pattern contours of the two beams generated at 19.7 and 30 GHz by the same feed.

Recently, the proposed operating principle has been experimentally validated in [26] by a 0.9 m parabolic reflectarray prototype that generates two spaced beams per feed at Tx and Rx frequencies using the reflectarray cell described in [20]. Figure 16 shows the prototype in the anechoic chamber and the 40.6 dBi pattern contours of the two measured beams generated simultaneously at 19.7 GHz and 30 GHz with a single dual-CP feed.

**Figure 17:**
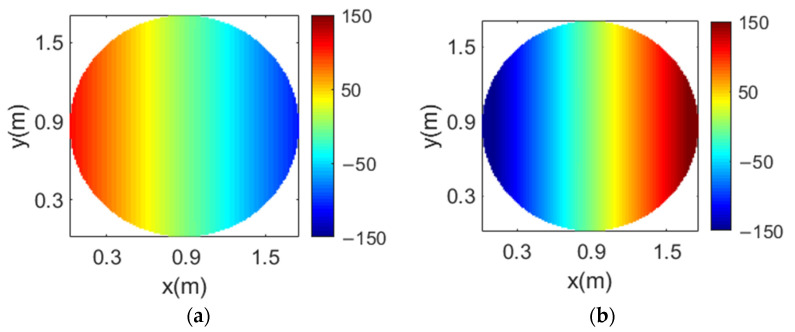
Required phase distributions of the 1.8 m parabolic reflectarray at the (**a**) lower and (**b**) upper operating frequencies for the same CP.

A 1.8 m parabolic reflectarray formed by 62,654 reflectarray cells with a period of 6.5 mm has been designed to deviate the beams in orthogonal CP ± 0.28° simultaneously at 19.7 and 29.5 GHz. Since the parabolic surface of the antenna focuses the beams radiated by the feeds, the required phase distributions on the reflectarray to split the orthogonal CP beams present a similar aspect as those of the subreflectarray shown in Section 4. Figure 17 shows the required phase distributions on the parabolic surface at 19.7 GHz and 29.5 GHz in one CP (the phase distributions in the orthogonal CP show the opposite phase variation to deviate the orthogonal CP beams in the opposite direction).

**Figure 18:**
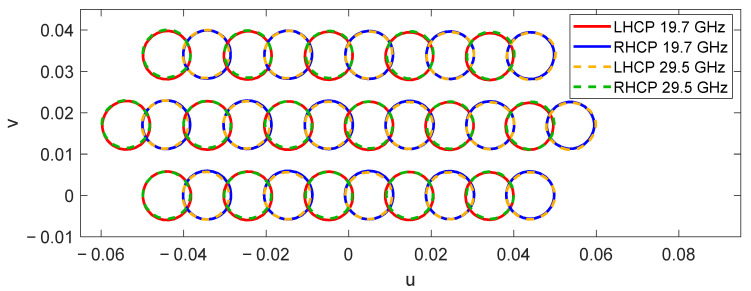
Contours of 32 beams generated simultaneously at 19.7 GHz and 29.5 GHz by the 1.8-m dual-band parabolic reflectarray illuminated by 16 feeds.

As in the first antenna solution shown in Section 3, the proposed dual-band parabolic reflectarray has been illuminated by the cluster of 27 feeds shown in Figure 3, and the simulations have considered three lines of feeds (16 feeds). Thus, the designed parabolic reflectarray is expected to generate 32 beams in orthogonal CP simultaneously in Tx and Rx. The simulated pattern contours of the 32 beams generated by the 1.8 m parabolic reflectarray at 19.7 and 29.5 GHz are depicted in Figure 18, where the contours of the beams at 19.7 and 29.5 GHz correspond to a gain level of 46 and 45 dBi, respectively. In a similar way to the previous dual reflectarray configuration, a second parabolic reflectarray operating at slightly different frequencies would produce the second half of the required four-color coverage.

**Figure 19:**
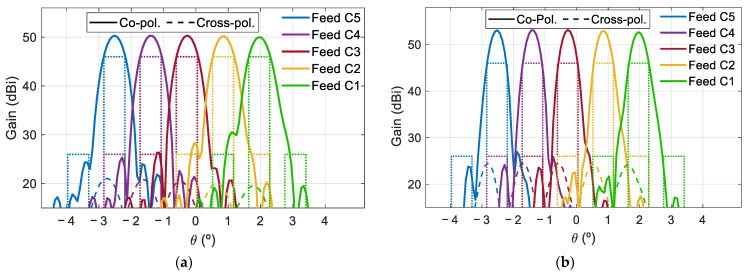
Cut of the simulated radiation patterns in v = 0 for the beams generated at (**a**) 19.7 GHz and (**b**) 29.5 GHz in same CP.

Figure 19 shows the horizontal cut in the plane v = 0 of the radiation patterns for the beams generated at 19.7 and 29.5 GHz. The main cuts of the radiation pattern have shown similar values of C/I to those of the previous configurations, with a XPD larger than 20 dB. Due to the large electrical size of the antenna at 29.5 GHz, the beams in Rx show a higher maximum gain than the beams in Tx; however, the shaping of the beams in Tx and Rx can be matched by the optimization procedure applied in [26].

In conclusion, the simulated results of the dual-band parabolic reflectarray have demonstrated a very satisfactory performance. The simulations show a suitable distribution of the beams with appropriate values of gain and XPD. As in the previous configurations, the C/I can be improved by a small increase of the antenna size to reduce the edge illumination levels on the reflectarray. Moreover, the reflectarray cell considered in the design makes it possible to implement additional optimization to shape the beams in Rx and to maintain the cross-polar levels in band, as in [26], by a proper adjustment of the dimensions and rotation angle of the elements [27].

## 6. Conclusions

In this paper, four novel antenna farms based on reflectarrays for broadband communication satellites in Ka-band have been proposed and evaluated. The exclusive characteristics of reflectarrays, which are able to operate independently at different frequencies or polarizations, have been applied in different ways to improve the SFPB operation compared to conventional reflectors. The impact of using flat or curved surfaces, single or dual-band operation, and single or dual antenna configurations for large reflectarrays in Ka-band has been assessed to achieve the best antenna performance. Table 2 summarizes the main characteristics of the proposed antenna solutions.

The preliminary simulations of single band reflectarrays to generate four beams per feed permits operation with a single antenna for Tx and an independent antenna for Rx, which simplifies the simultaneous operation in both bands as well as the design of the feed-chains. However, this solution involves a major difficulty in providing a stable performance in band due to the independent operation at close frequencies, requiring complex reflectarray cells to which intense optimizations must be applied. On the other hand, the two proposed dual-band reflectarrays show promising results. The simulations of the dual reflectarray system would require a further optimization of the subreflectarray to slightly reduce the cross-polar radiation, although the use of a dual configuration with two reflectarrays provides greater flexibility of operation than single aperture systems, which can be used to correct the shaping of the beams in the edge of the coverage. Finally, the dual-band parabolic reflectarray achieves an excellent antenna performance with a simple antenna configuration similar to that of the currently used reflectors. Moreover, the reflectarray elements can be optimized to properly shape the beams in Tx and Rx without the need to shape the antenna surface.

The core technologies of the proposed reflectarrays have been experimentally validated, which confirms the capabilities of reflectarrays to provide novel efficient multibeam antenna farms for broadband communications satellites in Ka-band. All the proposed antenna solutions make it possible to halve the number of antennas and feed-chains required onboard the satellite, from four reflectors to two reflectarray antennas, and from a total of N feed-chains (to produce a coverage of N spots) to N/2 feeds, allowing an important reduction in cost, as well as in mass and volume of the payload, which are severely limited in the satellite.

## Figures and Tables

**Table 1:** Antenna system requirements.

Parameter	Requirement
Number of spots	100
Reuse scheme	4 colors
Spot lattice	Triangular
Spot diameter	0.65°
Spot separation	0.56°
EOC gain	45 dBi
Single-entry C/I	20 dB
XPD	20 dB
Tx frequency band	19.2–20.2 GHz
Rx frequency band	29.0–30.0 GHz

**Table 2:** Main characteristics of the proposed antenna solutions.

Parameter	Flat Single-Band Reflectarray	Parabolic Single-Band Reflectarray	Dual-Band Dual Reflectarray	Parabolic Dual-Band Reflectarray
Antenna configuration	Single aperture	Single aperture	Dual configuration	Single aperture
Aperture type	Flat	Parabolic	Parabolic (main) + flat (sub)	Parabolic
Beams per feed	4	4	2	2
Frequency bands	1 (Tx or Rx)	1 (Tx or Rx)	2 (Tx and Rx)	2 (Tx and Rx)
Required antennas	2	2	2	2
Complexity of the cells	Very high	Very high	Low	Low
Operation in frequency	Very hard to implement	Hard to implement	Readily achievable	Readily achievable
Operation in CP	Hard to implement	Hard to implement	Readily achievable	Readily achievable
